# Occurrence of Polychlorinated Dibenzo-*p*-Dioxins and Dibenzofurans and Polychlorinated Biphenyls in Fruit and Vegetables from the “Land of Fires” Area of Southern Italy

**DOI:** 10.3390/toxics5040033

**Published:** 2017-11-10

**Authors:** Mauro Esposito, Antonella De Roma, Stefania Cavallo, Gianfranco Diletti, Loredana Baldi, Giampiero Scortichini

**Affiliations:** 1 Istituto Zooprofilattico Sperimentale del Mezzogiorno, 80055 Portici, Italy; antonella.deroma@izsmportici.it (A.D.R.); stefaniacavallo@izsmportici.it (S.C.); loredana.baldi@izsmportici.it (L.B.); 2 Istituto Zooprofilattico Sperimentaledell’Abruzzo e del Molise, 64100 Teramo, Italy; g.diletti@izs.it; 3 Istituto Zooprofilattico Sperimentale dell’Umbria e delle Marche, 06126 Perugia, Italy; g.scortichini@izsum.it

**Keywords:** dioxin, vegetables, Campania, Land of Fires, polychlorinated biphenyls

## Abstract

The concentrations of polychlorinated dibenzo-*p*-concentrations dioxins (PCDDs), polychlorinated dibenzofurans (PCDFs) (PCDD/Fs), and polychlorinated biphenyls (PCBs) were determined in fruit and vegetables collected in farms located in the well-known “Land of Fires” area of Southern Italy, in an effort to learn more about the environmental pollution of this high-risk area due to illegal waste dumping and uncontrolled burning near cultivated fields. Concentrations were in the range 0.011–2.26 ng g^−1^ for the six “indicator” non-dioxin-like PCBs (NDL-PCBs), and 0.0009–0.096 pg WHO toxic equivalent (TEQ) g^−1^ for the sum of dioxin-like PCBs (DL-PCBs) and PCDD/Fs. Lacking maximum limits for these contaminants in fruit and vegetables, the concentration values found were compared with the action levels set out in the EU Recommendations. These levels were never exceeded in the examined samples. In the present study, the highest mean value for PCDD/Fs + DL-PCB corresponded to apricots, olives, and nuts, while the lowest values were observed in endive and green beans. The results showed also that NDL-PCB levels in apricots were much higher than in any other food, suggesting that they can accumulate PCBs: this fruit might be proposed as a “sentinel” of the presence of these contaminants in the environment.

## 1. Introduction

Polychlorobiphenyls (PCBs), polychlorinated dibenzo-*p*-dioxins (PCDDs), and dibenzofurans (PCDFs) are organic chemical substances arisen from various anthropic activities, such as combustion processes and industrial manufacturing, with a strong toxicity due to their acute and chronic health effects on immune, nervous, endocrine, and reproductive systems, and their potential carcinogenic effects [1,2,3].

Since they show great resistance to chemical degradation and high lipophilicity that cause their accumulation in the food chain, there has been much attention paid to the presence of these organic pollutants in the diet. It was recently estimated that the average dietary exposure to dioxins and dioxin-like compounds in the Italian general population is close to the tolerable daily intake (TDI) of 2 pg WHO toxic equivalent (TEQ) kg^−1^ body weight [4], established by the Scientific Committee on Food (SCF) of the European Commission [5], and in a large part of the European population, PCBs intake is higher than recommended [6]. 

Human exposure to dioxins predominantly occurs via food intake [7]. The main contribution to the total intake originates from the consumption of animal fat [8]. Nevertheless, fruit and vegetables were estimated to contribute 13% of the total dietary intake, although the uncertainty was large [9]. They are important components of a healthy and balanced diet and represent a source of vitamins, minerals, and fibers and of a certain amount of energy (mainly in the form of sugar). Therefore, their consumption is constantly growing.

Knowledge of diet intake of certain persistent pollutants such as dioxins and PCBs therefore represents an important factor in ensuring the population’s health [10]. At the same time, vegetable products represent an important matrix for environmental quality bio-monitoring in high-risk areas such as those where waste incinerators and illegal burning of waste materials are active [11,12].

In the present study, a first detailed investigation relating to the concentrations of dioxins and PCB in fruits and vegetables from the Campania Region of Italy was performed. In the past decade, the interest in these substances has increased in the Campania Region after its lands were involved in illegal waste dumping phenomena and uncontrolled burning of wheels, plastics, textiles, and other industrial residuals along the roads bordering cultivated fields. The attention was paid in particular to that area between Napoli and Caserta sadly known as “Land of Fires,” which has been used for the illegal dumping, burning, and disposal of toxic waste and that houses a municipal waste incinerator.

This area is of interest due to past environmental pollution events that were discovered by finding high levels of dioxin in foods that were above permitted values [13,14,15]. 

To this aim, different fruits and vegetables were collected from the cultivated lands within the “Land of Fires” area between 2014 and 2016 in the proper seasonal period and analyzed for 17 polychlorinated dibenzo-*p*-dioxins and dibenzofurans (PCDD/Fs), 12 congeners of dioxin-like polychlorobiphenyls (DL-PCBs), and 6 non-dioxin-like polychlorobiphenyls (NDL-PCBs).

While there is sufficient data available on the levels of PCDDs, PCDFs, and PCBs in foods of animal origin, there is much less on their levels in fruit and vegetables [4,16], and this makes it difficult to estimate the exposure of the population.

Mainly due to low levels of dioxins in vegetables, the determination of the average content in samples resulted in an estimate with substantial uncertainty. Therefore, the present study was conducted based on composite samples for all relevant types of fruit and vegetables.

## 2. Materials and Methods 

### 2.1. Selection of Vegetables and Sampling Area 

Based on the typical plantation of the Campania region lands, 16 fruits and 26 vegetables were selected (Table 1): below-ground growing vegetables (onion, garlic, fennel, potato, and turnip), above-ground growing vegetables and fruits (cauliflower/kohlrabi, zucchini, artichoke, broccoli, turnip tops, and strawberries), leafy vegetables at ground level (lettuce, cabbage, chicory, endive, and radish) and vegetables and fruits growing far above ground level (green bean, pea, broad bean, wheat, corn, pepper, tomato, aubergine, chili pepper, apple, grain, apple, apricot, cherry, plum, peach, pear, clementine, mandarin, lemon, orange, kaki, grape, walnut, and hazelnut). 

**Figure 1:**
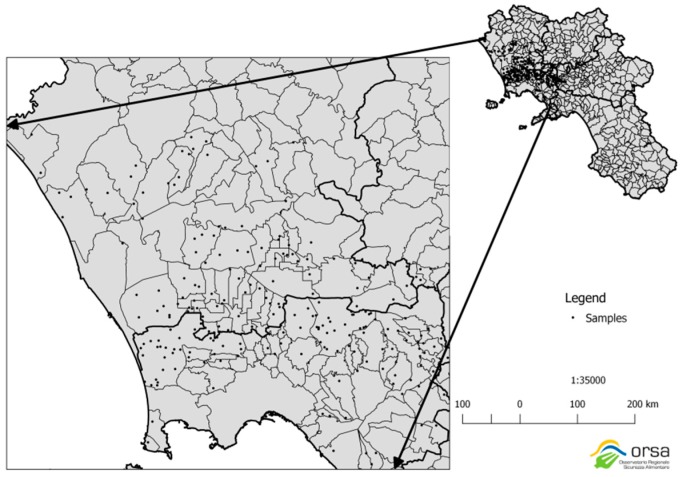
Map showing the zones of sampling sites of vegetables and fruit samples from the “Land of Fires.”

Samples were collected in their specific growing period between 2014 and 2016 from orchards or vegetable plantations located in different parts of the “Land of Fires,” in the Campania Region, mainly where there are some potential sources of contamination, such as an incinerator or waste burning. In particular, this area is located between the Domizio-Phlegrean Coast, the Agro Aversano-Atellano lands, the districts of Acerra and Nola, the lands around Vesuvius, and the city of Naples, affecting a total of 95 municipalities in the provinces of Benevento, Caserta, and Naples (Figure 1).

As established by the sampling procedures [17], a hypothetical “X” on the agricultural identified area was drawn and along the lines and individual samples, of at least 1 kg, per hectare were collected depending on the size of the ground. The individual samples formed the composite sample, whence the laboratory sample was prepared.

Samples were collected by qualified staff wearing vinyl gloves, put into zip bags, and immediately sent to the laboratory. Before analysis, adherent matter such as soil, foul parts, and non-edible leaves and stems were removed manually and samples were washed. From each kind of fruit or vegetable, individual samples were combined to make a pooled sample. Other samples and the remainder of the individual samples were stored at −20 °C and available for further investigations.

### 2.2. Chemicals and Standards

All solvents (toluene, n-hexane and dichloromethane) were of ultra-trace analysis grade and were purchased from Sigma-Aldrich (Milan, Italy). Pre-packed multi-layer silica, alumina, and carbon columns were obtained from a Fluid Management System (Waltham, MA, USA). All standard solutions were supplied by Wellington Laboratories (Toronto, ON, Canada). Before analysis, all samples were spiked with a mixture of ^13^C_12_-labeled internal standard (IS) of DL-PCBs (IUPAC Nos. 77, 81, 105, 114, 118, 123, 126, 156, 157, 167, 169, and 189), six indicator NDL-PCBs (IUPAC Nos. 28, 52, 101, 138, 153, and 180), and PCDD/Fs (2,3,7,8 chlorine-substituted congeners) (Wellington Laboratories, Guelph, ON, Canada).

### 2.3. Sample Processing and Analysis

The sample extraction and clean-up were carried out as previously reported [18]. The fraction containing DL-PCBs and NDL-PCBs was collected after elution from the alumina column, while the fraction containing PCDD/Fs was eluted from the carbon column. The two fractions were concentrated, first under vacuum and then under nitrogen, and the remainders were dissolved in the corresponding recovery standards solutions (^13^C_12_-labeled congeners).

The HRGC/HRMS measurements were carried out using a GC Trace Series 2000 coupled with an MAT 95 XP (Thermo Fisher, Bremen, Germany). The congeners of PCDD/Fs were separated by high resolution gas chromatography (HRGC) on a DB-5 MS capillary column (60 m × 0.25 mm, 0.10 µm film thickness, J & W Scientific, Folsom, CA, USA) and determined by high resolution mass spectrometry (HRMS). The congeners of DL-PCBs and NDL-PCBs were separated by HRGC on an HT-8 capillary column (60 m × 0.25 mm, 0.25 μm film thickness, SGE Analytical Science Pty, Ltd., Victoria, Australia) and determined by HRMS. 

### 2.4. Quality Control Criteria

Samples were tested by a validated method routinely used for PCDD/Fs and PCBs analysis in food and feed and successfully tested in a number of inter-laboratory studies. The method used was an in-house adaptation of the US EPA Methods [18].

PCDD/Fs and DL-PCBs TEQ values were calculated using the World Health Organization Toxic Equivalency Factors (WHO-TEFs2005). The upperbound (UB) concentrations were calculated assuming that all values of the different congeners less than the limit of quantification were equal to the limit of quantification and were expressed in pg-TEQ/g fresh weight. The sum of six indicator congeners was calculated for NDL-PCBs as UB concentration, and expressed as ng/g.

A laboratory blank and a control sample were analyzed for each batch of 10 and 20 samples, respectively. Recovery rates of labeled congeners ranged from 60% to 90%, and the analytical uncertainty was in the order of ±20% for WHO-TEQs and the sum of six NDL-PCBs.

Results were also analyzed through a determination of Pearson correlation coefficients and principal component analysis (PCA) using the Minitab Statistical Software by Minitab Company.

## 3. Results and Discussion

A total number of 228 samples were collected from cultivated fields located in the so-called “Land of Fires” area in the Campania Region. The concentrations (upper bound, on a fresh weight basis) of WHO-PCDD/F-TEQs, WHO-PCB-TEQs, and Σ6PCB found in the respective fruit and vegetable items were summarized in terms of minimum, mean, median, and maximum in Table 2. Fruits and vegetables in Table 2 are mainly identified individually, with the exception of some family groupings in which there were only a few samples of species therein.

In the analyses of vegetables, extremely low detection limits were obtained (<1 pg/g fresh weight for each congener). As regards the NDL-PCB, the mean content value obtained for all samples was 0.17 ng/g fresh weight with a range from 0.01 to 2.6 ng/g. 

Between fruits, apricots showed a mean concentration higher than any other vegetables analyzed: mean concentration was 0.91 ng/g with values ranging from 0.003 to 1.56 ng/g. However, the highest value of concentration was detected in one sample of hazelnut (2.6 ng/g), and in general nuts (hazelnut, walnut) showed similar levels of NDL-PCBs (mean 0.44 ng/g).

Between vegetable species (*Brassicaceae*, *Solanaceae*, etc.), kohlrabi (roots) revealed a high value for the sum of NDL-PCBs (mean 1.26 ng/g). This has been predicted and observed before since it has a large surface/content ratio that perfectly absorbs hydrophobic contaminants from soil, and for its extremely long presence on the field (9 months) [19].

High values with respect to other vegetables were found in olives (mean content 0.52 ng/g): this result can be explained by their high fat content and hence the accumulation availability of these lipophilic contaminants.

The lack of maximum limits as well as action levels for NDL-PCB makes it difficult to assess the risk associated with their levels in vegetables grown in the Campania Region; however, they can be compared with the literature data, even if such data are scarce.

The overall median and mean Σ6PCB level amounted to 0.055 and 0.174 ng/g, respectively, and is relatively higher than that obtained in a similar background contamination study in Germany (0.054 and 0.072 ng/g, respectively) [20]. On the contrary, our concentrations of the six “indicator” NDL-PCBs are lower than those found in the fruit and vegetables harvested in different parts of the Mantua district in Northern Italy, ranging from 0.01486 to 4.504 ng/g [10]. In general, we can also confirm that Congener 153 was the most abundant and recurrent in the vegetable samples, as confirmed by Grassi [10]. In pumpkins, courgettes, and peas, there is also a significant abundance of Congener 138. On the contrary, in most fruit samples, a higher concentration was found for low-chlorinated Congeners 28 and 52.

With regard to the PCDD/F content, the results of analyses revealed that most of the congeners were measured below the limit of quantification (LOQ).

Therefore, the sum values are very low compared to action levels fixed for DL-PCBs and PCDD/Fs by Commission Recommendation 2014/663/EU and to those reported in the literature for vegetables collected in highly polluted areas [21,22,23,24].

Since soil–plant transfer of PCDD/Fs was found to be highest in the cucumber family (Cucurbitaceae) [25], the relatively high levels in the melon sample was not surprising: between samples belonging to this family, our level for melon (0.021 pg/g) was similar to the mean value (0.020 pg/g) obtained in the courgette samples reported by Breitweg-Lehmann [20]. 

As regards DL-PCBs, in general, LOQ amounts higher than those of Congeners 81 and 169 were obtained only in 1.3% and 6.0% of cases, while Congeners 118, 105, and 77 were more abundant and recurring in the vegetable samples. In particular, Congeners 118 and 105, characterized by low chlorination, were found in all vegetable samples, and the presence of these more volatile congeners led to the hypothesis of a mechanism of accumulation based on the re-suspension of PCBs in air from the soil with subsequent deposition on the vegetables [10]. However, the low TEF values of these substances ensure that their contribution to the TEQ could be very low.

Taking the DL-PCB content in the different vegetable species into consideration, in confirmation of that already observed for the six indicator PCBs, only wheat, melon, apricots, kohlrabi, and walnuts showed significant concentration values. In particular, melon exhibited the highest mean lowerbound value for ∑DL-PCBs on fresh weight (0.056 pg TEQ/g), followed by kohlrabi (0.049 pg TEQ/g), wheat (0.045 pg TEQ/g), apricots (0.043 pg TEQ/g), and walnuts (0.028 pg TEQ/g). 

**Figure 2:**
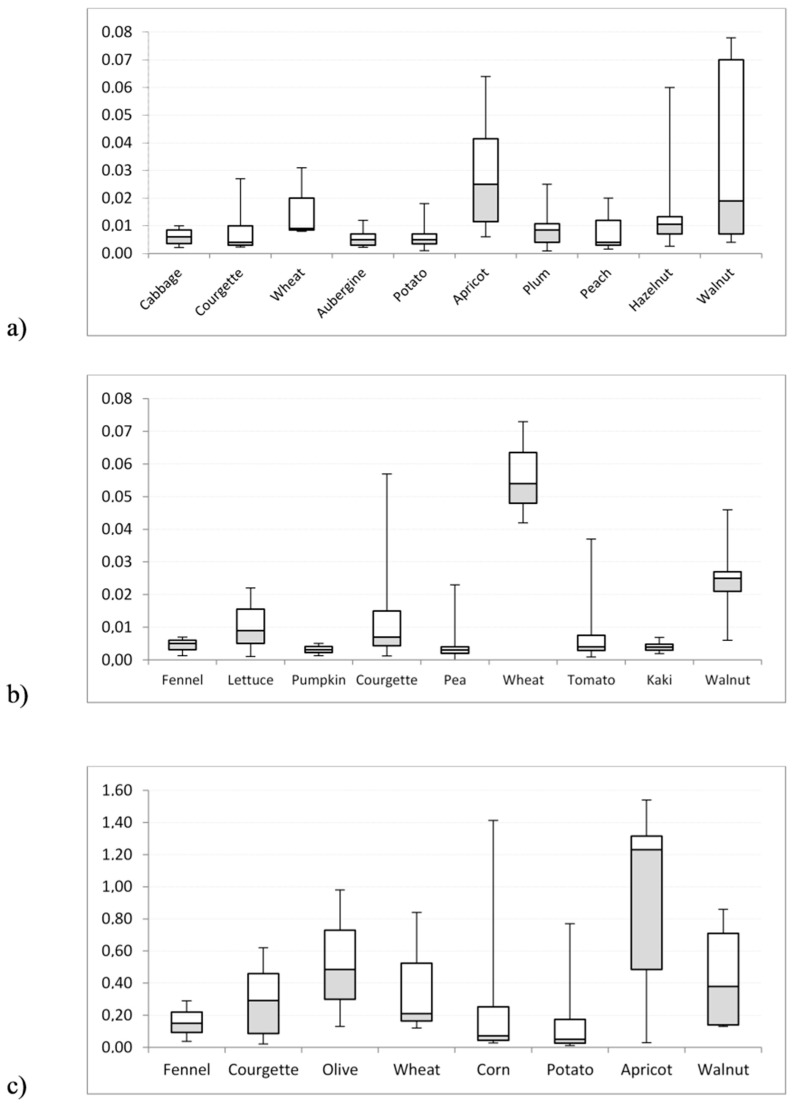
Box–whisker plots of median concentrations of PCDD/Fs (**a**), DL-PCBs (**b**), and NDL-PCBs (**c**) in vegetable samples collected in the “Land of Fires” (Campania, Italy). Values are expressed as pg TEQ/g for PCDD/Fs and DL-PCBs, and as ng/g for NDL-PCBs.

For the latter product, the higher fat content might explain a higher accumulation. Besides nuts, compared to other plant species, a high content of microelements has also been observed in the same area [26]. Figure 2 shows box-and-whisker plots for selected samples. They are used to show overall patterns of response for a group and provide a useful way of visualizing the range of concentration. For each analyte, the plots show a strong variability of the dataset distribution.

**Figure 3:**
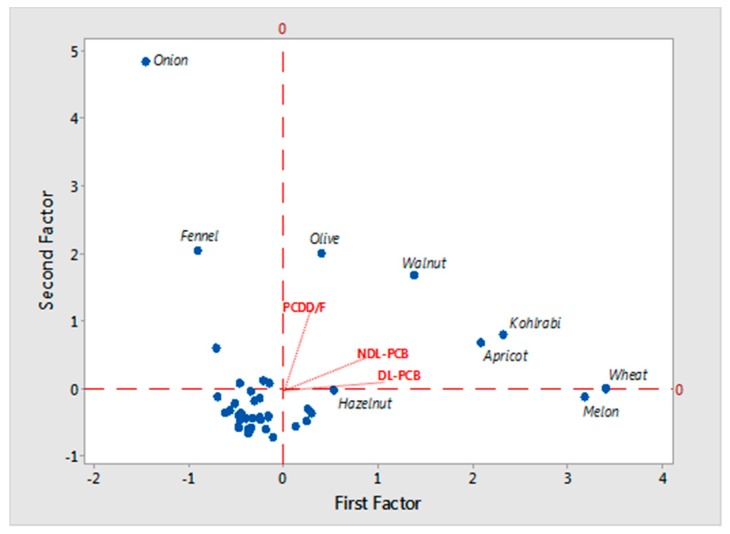
Biplot of PC1 and PC2 for PCDD/Fs, DL-PCBs, and NDL-PCBs in all samples.

A multivariate statistical analysis of the data was assessed through the PCA, as shown in Figure 3.

PCA estimates the correlation structure of the variables by finding hypothetical new variables (principal components (PCs)) that account for as much of the variance (or correlation) as possible in a multidimensional data set. These new variables are linear combinations of the original variables [27]. The significant PCs, also called only factors, are linear combinations of the observed variables, with VARIMAX normalized rotation, and were selected according to the Kaiser criterion [28]. The biplot shows the samples analyzed in the present study in the space of the first and second principal components, which take into account 40.20% and 56.78% of variability of the data, respectively. This biplot represents the observations and variables simultaneously in the space and visually illustrates the associations among these contaminants in all samples from the Campania Region. 

In this plot, olive, apricot, kohlrabi, hazelnut, and walnut samples were mainly located in the positive direction of the first principal component, whereas the majority of the other samples were restricted in the axis center, in the negative direction of the two principal component, revealing their reduced influence on the reference sample group. Thus, results of the PCA indicated that the PCB congener profile of apricots and kohlrabi were richer in NDL-PCBs than the other samples. Table 3 shows the factor loadings of PCDD/Fs, DL-PCBs and NDL-PCBs from the PCA. The factor loadings, also called component loadings in PCA, are the correlation coefficients between the original variables (rows) and factors (columns).

In the interpretation of PCA patterns, factor loadings greater than 0.71 are defined as excellent, while those less than 0.32 are regarded as poor [29]. Communality values, the proportion of variability of each variable explained by the factors, can be especially useful in comparing fits. Their values should be close to one, as observed in our case. This indicates that the model explains most of the variation of those variables.

Exposure to the contaminants under investigation through vegetable and fruit intake was assessed by combining typical Italian consumption data for children and adults [30] with the average concentrations we found. Results were expressed as percentages of the tolerable daily intake (TDI) and of the minimal risk level (MRL), respectively, for ∑TEQ (PCDD/Fs+DL-PCBs) and ∑6NDL-PCBs, taking into account the reference limits established by the Scientific Committee on Food of the European Commission [5] for the TDI of dioxin-like compounds (2 pg TEQ kg^−1^ bw/day) and the Agency of Toxic Substances and Disease Registry [31] for the MRL of total PCBs (20 ng kg^−1^ bw/day). 

The standard deterministic method to estimate exposure can be described according to Equation (1) for an individual *i*:

*E_i_* = (∑ *C_ij_T_j_*)/*w_i_* (1)

where *w_i_* is the individual body weight of the individual I; *C_ij_* is the consumption of food *_j_* by the individual I; *T_j_* is the average PCDD/F or DL-PCB contamination of food *_j_*.

The average intake of contaminants was estimated by multiplication and summation of the average consumption of the various vegetables per capita with the concentrations in these vegetables. It appears that the total intake related to PCDD/Fs and DL-PCBs varies from 0.00021 in children (3–10 years) for the consumption of vegetables to 0.00103 pg TEQ kg^−1^ bw/day in adults for the consumption of fruit. NDL-PCB intake ranged from 0.00687 in adults for the consumption of vegetables to 0.0564 ng kg^−1^ bw/day in children (3–10 years) for the consumption of fruit. 

The contributions to the total intake from potatoes were calculated separately because of their high consumption. Dioxin concentrations ranged from 0.00063 pg TEQ kg^−1^ bw/day in children to 0.00021 pg TEQ kg^−1^ bw/day in adults; NDL-PCB concentrations ranged from 0.0147 ng kg^−1^ bw/day to 0.00636 ng kg^−1^ bw/day. Therefore, we can affirm that the low concentrations of these substances ensure a low exposure of these substances to consumers of the vegetables and fruits produced in the studied area. 

## 4. Conclusions

Dioxins and PCBs in soil and in atmosphere may be the source of these pollutants in the vegetables growing in this environment. In this study, carried out in the cultivated field located in the so-called “Land of Fires” in the Campania Region of Southern Italy, the results showed a very low contamination level in vegetables.

The low levels of dioxins and PCBs found in plants demonstrate that these matrices poorly accumulate these types of pollutants and thus that the degree of environmental pollution by dioxins and PCBs in the Land of Fires is low, as demonstrated by recent results obtained in animal matrices [32,33]. The results, in turn, show, in the consumption of vegetables and fruits produced by the population in this area, a low dietary exposure of these contaminants.

## Figures and Tables

**Table 1:** Fruit and vegetable samples analyzed in this study.

*Fruits*	*Group*	Sample Size *
Ebenaceae	kaki	2
Vitaceae	grape	4
Juglandaceae	walnut	5
Betulaceae	hazelnut	16
Rosaceae	strawberry, apple, apricot, cherry, plum, peach, pear	50
Rutaceae	clementine, mandarin, lemon, orange	8
***Vegetables***	***Group***	Sample Size *
Cucurbitaceae	watermelon, melon, pumpkin, courgette	11
Amaryllidaceae	onion, garlic	3
Apiaceae	fennel	3
Compositae	lettuce	3
Asteraceae	chicory, endive, artichoke	5
Oleaceae	olive	6
Fabaceae	green bean, pea, broad bean	9
Poaceae	wheat, corn	15
Brassicaceae	broccoli, cabbage, kohlrabi, turnip tops, turnip, radish	13
Solanaceae	pepper, tomato, aubergine, potato, chili pepper	75
**Total**		**228**

* Referred to the composite sample, of at least 1 kg.

**Table 2:** Mean and standard deviation of dioxin-like polychlorinated biphenyls (DL-PCBs), polychlorinated dibenzo-*p*-dioxins and dibenzofurans (PCDD/Fs), and non-dioxin-like polychlorinated biphenyls (NDL-PCBs) reported as upperbound concentrations.

	DL-PCB (pg WHO-TEQ/g)				PCDD/F (pg WHO-TEQ/g)				NDL-PCB (ng/g)			
	min	mean	median	max	min	mean	median	max	min	mean	median	max
***Vegetables***												
Amaryllidaceae (3) *	0.001	0.003	0.004	0.004	0.003	0.025	0.003	0.069	0.019	0.056	0.040	0.110
Fennel (3)	0.001	0.004	0.005	0.007	0.004	0.036	0.007	0.096	0.037	0.159	0.150	0.290
Asteraceae (5)	0.001	0.007	0.008	0.013	0.003	0.007	0.007	0.012	0.023	0.063	0.074	0.087
Brassicaceae (13)	0.002	0.008	0.006	0.051	0.002	0.010	0.008	0.034	0.026	0.166	0.064	1.260
Lettuce (3)	0.001	0.011	0.009	0.022	0.003	0.004	0.004	0.005	0.079	0.089	0.080	0.108
Pumpkin (2)	0.001	0.003	0.003	0.005	0.004	0.005	0.005	0.006	0.034	0.046	0.046	0.058
Courgette (7)	0.001	0.015	0.007	0.057	0.002	0.008	0.004	0.027	0.022	0.293	0.291	0.620
Fabaceae (9)	0.004	0.004	0.004	0.004	0.002	0.002	0.002	0.002	0.034	0.070	0.070	0.106
Olive (6)	0.003	0.024	0.021	0.055	0.008	0.040	0.050	0.072	0.130	0.522	0.485	0.980
Wheat (3)	0.042	0.056	0.054	0.073	0.008	0.016	0.009	0.031	0.120	0.390	0.210	0.840
Corn (12)	0.001	0.013	0.006	0.066	0.004	0.006	0.006	0.012	0.028	0.229	0.072	1.413
Peppers (8)	0.001	0.004	0.004	0.008	0.003	0.006	0.006	0.008	0.033	0.051	0.020	0.095
Tomato (31)	0.001	0.007	0.004	0.037	0.001	0.005	0.004	0.020	0.019	0.049	0.042	0.120
Aubergine (13)	0.001	0.004	0.003	0.007	0.002	0.006	0.005	0.012	0.019	0.046	0.042	0.101
Potato (23)	0.001	0.008	0.005	0.028	0.001	0.006	0.005	0.018	0.012	0.140	0.051	0.770
***Fruits***												
Rosaceae (6)	0.002	0.004	0.003	0.006	0.002	0.007	0.005	0.019	0.011	0.064	0.026	0.230
Apricot (7)	0.003	0.045	0.034	0.086	0.006	0.029	0.025	0.064	0.029	0.914	1.230	1.540
Plum (22)	0.001	0.007	0.006	0.023	0.001	0.008	0.009	0.025	0.012	0.050	0.040	0.098
Peach (15)	0.001	0.006	0.005	0.023	0.002	0.008	0.004	0.020	0.017	0.041	0.038	0.067
Rutaceae (8)	0.008	0.003	0.001	0.008	0.003	0.006	0.006	0.008	0.028	0.065	0.037	0.024
Grape (4)	0.002	0.009	0.009	0.017	0.004	0.012	0.007	0.032	0.140	0.158	0.150	0.190
Melons (2)	0.007	0.032	0.032	0.058	0.001	0.011	0.011	0.021	0.039	0.534	0.534	1.030
Hazelnut (16)	0.003	0.020	0.008	0.106	0.003	0.014	0.011	0.060	0.049	0.445	0.185	2.600
Kaki (2)	0.002	0.004	0.004	0.006	0.002	0.003	0.003	0.003	0.036	0.043	0.043	0.050
Walnut (5)	0.006	0.035	0.030	0.078	0.004	0.036	0.019	0.078	0.130	0.444	0.380	0.860

* Sample size. TEQ: toxic equivalent.

**Table 3:** Rotated factor loadings and communalities after Varimax rotation.

Variable	Factor 1	Factor 2	Communality
DL-PCB	0.976	0.207	1.00
PCDD/F	0.222	0.969	1.00
NDL-PCB	0.813	0.295	1.00
